# Use of a Non‐Endoscopic Capsule‐Sponge Triage Test for Reflux Symptoms: Results From the NHS England Prospective Real‐World Evaluation

**DOI:** 10.1111/apt.18472

**Published:** 2025-01-10

**Authors:** Vlasios Gourgiotis, Charlotte Graham, Kristen Foerster, NHS England Pilot and NHS England Oversight Group, Rebecca C. Fitzgerald, Rory Harvey, Danielle L. Morris

**Affiliations:** ^1^ Barts Cancer Institute Queen Mary University of London London UK; ^2^ NHS England England UK; ^3^ Early Cancer Insitute Department of Oncology, University of Cambridge Cambridge UK; ^4^ Bedfordshire Hospitals NHS Foundation Trust Bedfordshire UK; ^5^ Department of Gastroenterology East and North Hertfordshire NHS Trust Hertfordshire UK

**Keywords:** Barrett's oesophagus, biomarkers, capsule sponge test, endoscopy, GERD or GORD, non‐endoscopic test

## Abstract

**Background:**

Acid reflux is a common presentation in primary care leading to a high volume of referrals to endoscopy that are often normal.

**Aims:**

To determine whether a non‐endoscopic capsule sponge biomarker test could triage patients with low‐risk reflux symptoms, reduce endoscopy waiting lists and identify Barrett's oesophagus in a real‐world setting.

**Methods:**

Patients with reflux symptoms on NHS endoscopy waiting lists who were offered a capsule sponge (test group) between February 2021 and August 2022 were included in this national multicentre prospective cohort study and compared with eligible patients on the standard endoscopy pathway (counterfactual group).

**Results:**

Two thousand one hundred seventy patients from 23 hospitals undertook a capsule sponge triage test and submitted follow‐up data, of whom 1694 (78%) were discharged without endoscopy and 476 (22%) were referred for endoscopy. In a detailed subgroup analysis 1411/1549 (92%) attended and successfully swallowed the capsule of whom 307/1411 (21.8%) required endoscopy. Of 111 patients with positive capsule sponge biomarker results who had endoscopy, 19.8% had Barrett's oesophagus. In comparison, in patients with negative sponge biomarkers who were referred for endoscopy due to ongoing symptoms, none had Barrett's oesophagus (*p* = 0.0002). Eighty‐two percent surveyed (267/357) were satisfied with the alternative pathway.

**Conclusions:**

Capsule sponge triage is feasible, safe and acceptable. It substantially reduces the endoscopy burden for routine reflux referrals with a favourable diagnostic yield for Barrett's oesophagus. Longer term follow‐up will help to confidently assess the impact of the programme and place of capsule sponge in the diagnostic pathway.

## Graphical abstract

Graphical representation of our findings investigating whether a capsule sponge biomarker test could triage patients with low‐risk reflux symptoms, reduce endoscopy waiting lists and identify Barrett's oesophagus in a real‐world setting.

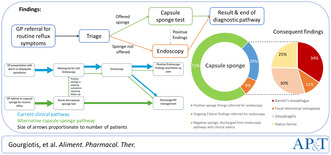

## 1 Introduction

Gastro‐oesophageal reflux disease (GORD) is common [1, 2] and while 60% have no oesophageal damage, severe reflux may cause ulceration, stricture and Barrett's oesophagus (BO), which can progress to oesophageal adenocarcinoma [3, 4].

From health‐service perspectives, routine referrals for reflux contribute to long endoscopy and pathology waiting lists, impacting more urgent and suspected cancer referrals. Physicians face a conundrum since most endoscopies are normal [5], while most individuals with BO are undiagnosed [6]. Considering poor UK cancer outcomes, the NHS Long Term Plan aims for improvement through early diagnosis and treatment [7]. Therefore, more efficient measures to control endoscopy waiting lists and prioritise individuals likely to have significant pathology are required.

The NHS Cancer Programme identified capsule sponge (CS), a non‐endoscopic test involving oesophageal cell collection for cytopathology and immunostaining, as a potential triage tool for reflux and instigated a prospective cohort study during the pandemic. This evaluation sought to determine whether CS could reduce endoscopy referrals and support earlier diagnosis. Several trials in which patients had a capsule‐sponge followed by an endoscopy (totalling > 2000 procedures) show that CS is well‐tolerated and TFF3 immunohistochemical biomarker identifies BO with accuracy (80%–89% sensitivity and 92% specificity) [8, 9, 10, 11]. A large‐scale randomised trial of CS in 13,000 patients showed 10x more BO diagnoses compared to general practitioner (GP) usual care [12]. The addition of p53 and atypia can be used to evaluate malignant progression (*n* = 891 procedures with CS and endoscopy) although the previous studies have focussed on patients undergoing Barrett's surveillance in whom the performance characteristics may differ compared with this routine reflux population with a very low cancer risk [13]. It is also possible to identify other inflammatory conditions from a CS sample [14].

In this prospective cohort study, we performed CS with TFF3, p53 and atypia biomarkers in hospitals across England between February 2021 and August 2022 to determine impacts on endoscopy demand, patient outcomes, patient experience and cost‐effectiveness.

## 2 Methods

### 2.1 Evaluation Cohorts

**FIGURE 1:**
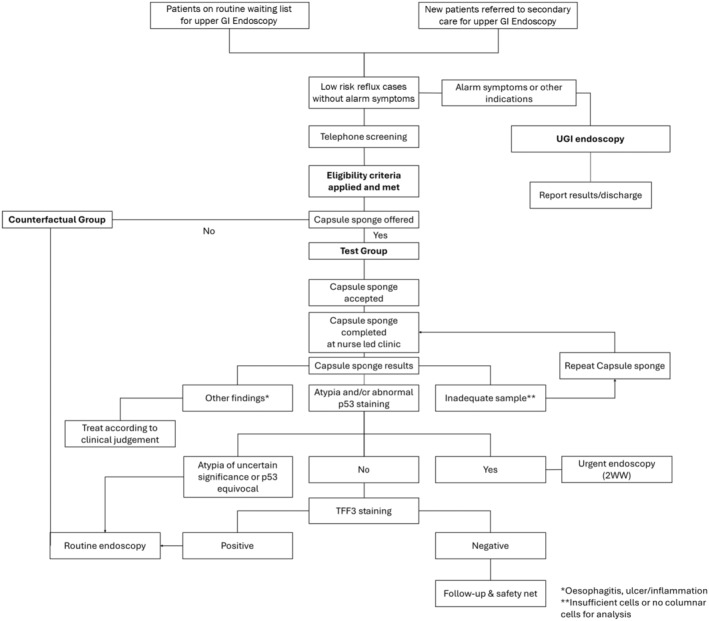
Decision tree used by the clinical teams to enrol patients into the capsule sponge prospective cohort study and to manage their ongoing clinical care according to the results. Patients who were eligible for the capsule sponge but who were not offered the test were included in a counterfactual group. 2WW, 2 week wait; TFF3, Trefoil factor 3; UGI; upper gastrointestinal.

Thirty hospitals across England were initially selected to take part in delivering non‐endoscopic CS for patients with reflux symptoms of whom 23 agreed to return management outcomes. Patients referred on routine endoscopy pathways, with no alarm symptoms, between February 2021 and August 2022 were eligible. The evaluation was part of a larger pilot programme that ended in March 2024. A counterfactual group on routine endoscopy referral pathway, within the same period, was formed. There was no randomisation. If patients were ineligible for CS or declined, they were excluded to avoid bias. NHS England appointed an oversight committee who developed guidance (Data S1, NHS England Clinical Guidance), reviewed progress and appointed IQVIA, a health research services consultancy, to assist with evaluation. The oversight panel included patient and public representatives. All patients signed NHS consent forms and only completely anonymised and aggregated data were provided in keeping with national Public Benefit and Privacy Panel (PBPP) approval from the Caldicott guardian. A summary of pathways and identification of counterfactual patients are shown in Figure 1 and comparative subgroup demographics for which full data were available are shown in Table 1.

**TABLE 1:** Clinical and demographic data of capsule sponge subgroup.

Characteristic	Capsule sponge group (*N* = 1549)
Age at referral (median, IQR)	52 (40–62)
Male	656 (42.3%)
Time between referral and index date (days; median, IQR)	27 (13–70)
Ethnicity	
White	1245 (80.4%)
Non‐White^a^	304 (19.6%)
Type of referral	
Direct access	246 (15.9%)
Routine	1303 (84.1%)
Weight loss	
Yes	43 (2.8%)
No	1506 (97.2%)
Heartburn	
Yes	229 (14.8%)
No	1320 (85.2%)
Waterbrash	
Yes	14 (0.9%)
No	1535 (99.1%)
Reflux	
Yes	1150 (74.2%)
No	399 (25.8%)
Use of acid suppressants within the last 6 months	
Yes	1303 (84.1%)
No	209 (13.5%)
Missing	37 (2.4%)
IMD^b^ quintile (most‐least deprived)	
1	284 (18.3%)
2	323 (20.9%)
3	289 (18.7%)
4	307 (19.8%)
5	326 (21.1%)
Missing	20 (1.3%)

^a^ Non‐white includes Asian, Black American or African American, Hawaiian or Other Pacific Islander, American Indian. As the numbers in each category are small these are grouped together.

^b^ IMD is Indices of Multiple Deprivation with quintile 1 being the most deprived and 5 being the least deprived. IQR: Interquartile range.

### 2.2 CS and Endoscopy Procedures

**FIGURE 2:**
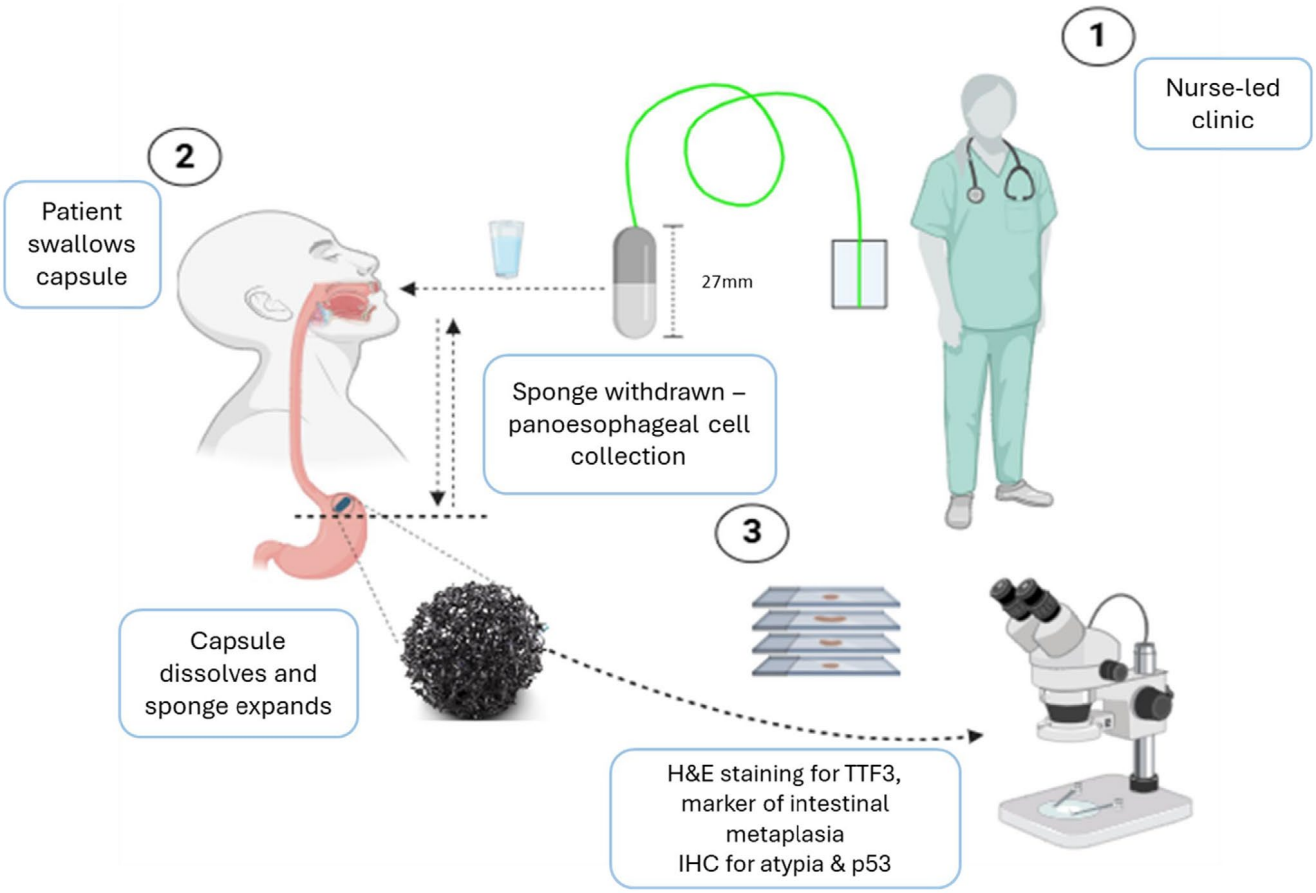
Schematic illustration of the capsule sponge procedure. The procedure is carried out in a nurse‐led clinic, where the patient is consented. The patient swallows the capsule/pill with water while the nurse holds the other end. After 5–7 min, after the pill has dissolved thanks to the gastric juices and the sponge has fully expanded, the nurse pulls the sponge out. This allows for pan‐oesophageal cell collection, which is then taken to the laboratory taken to the laboratory for IHC staining for TFF3, marker of intestinal metaplasia, H&E and IHC for atypia and p53. The whole procedure takes around 10 min to complete.

The CS test comprises a capsule tethered to a thread, and it is conducted as an office‐based test with the patient in the sitting position. The CS starts off as a capsule, attached to a thread. When a patient swallows the capsule with a glass of water and it reaches the stomach, the coating dissolves and the sponge inside it expands. After 7 min to allow time for full expansion of the sponge, it is withdrawn through the mouth by pulling on the thread. As it is withdrawn, the sponge collects cells from the gastric cardia and the entire length of the oesophagus (Figure 2).

The CS service was nurse‐run with consultant oversight. The nurse checked eligibility (see clinical guidance in Data S1) by telephone to ensure no contra‐indications and explain the pathway. Nurses were instructed to ensure no alarm symptoms requiring urgent endoscopy because this is often not clear on the referral form, which did not have CS as an option at the time of this study.

*Inclusion criteria for cytosponge*:

Patients with symptoms of reflux including:

- Heartburn (burning sensation on chest usually after eating).
- Regurgitation (an unpleasant sour taste in mouth caused by stomach acid).
- Waterbrash (excessive salivation).

*Exclusion criteria for Cytosponge (absolute contraindications)*:

- Alarm symptoms:
  ○ dysphagia
  ○ dyspepsia and weight loss
  ○ dyspepsia and anaemia
- Previous cancer of the oesophagus.
- Patient with a diagnosis of an oropharyngeal, oesophageal or gastro‐oesophageal tumour.
- Patient who has had treatment to the oesophagus for example, photo dynamic therapy, endoscopic mucosal resection, radio frequency ablation, surgery.
- Patient known to have oesophageal varices or cirrhosis of the liver.
- Patient with a known anomaly of the oesophagus for example, webbing, pouch, stricture and so forth.
- Patients who are pregnant (relative contraindication, Cytosponge not harmful but may not be appropriate).
- Patients unable to give consent
- Patients who have had a stroke or any other neurological disorder where their swallowing has been affected.
- Patients who have had a myocardial infarction in the last 3 months.

*Patients to consider as having relative contra‐indications to Cytosponge use*:

- Patients who have had fundoplication may be candidates for Cytosponge but may have reflux symptoms post procedure.

The CS (Cytosponge, Medtronic Inc.) procedure was carried out by NHS nurses. Training was provided via online webinar and live training from a trainer with sign‐off required for independent practice. Due to low aerosol rates [15], procedures could be carried out in outpatient consulting rooms. Following removal, the sponge was placed in BD SurePath Preservative Fluid and sent to Cyted Ltd. centralised laboratory (Huntingdon, UK) for processing to paraffin blocks (FFPE) and cytopathology reporting according to ISO 15189 standards [16].

Sample adequacy was considered if columnar cells were present, confirming proximal stomach was sampled. If no columnar cells were present the patient was offered repeat testing [12]. Atypia (including dysplasia and atypia of unknown significance) was assessed on superficial and deep sections processed to a Haematoxylin and Eosin (H&E) slide. TFF3 immunohistochemistry (Roche 7F1.21 clone) and p53 immunohistochemistry (Roche Bp53‐11 clone) was performed on adjacent superficial and deep subsequent FFPE slides. p53 staining with intensity of three considered significant. Samples with suspected atypia or aberrant p53 expression were reviewed by a second pathologist, as is best practice [16]. A digital platform reported results.

The pathology report included recommendations for endoscopy, if indicated and urgency. Clinicians were advised to treat results in clinical context as gastric pathology cannot be assessed. Thus, for negative samples if clinicians had concerns referrals were encouraged to endoscopy or other investigations. Hospitals communicated with patients to convey results according to local preferences for example, telephone, clinic or letter. Some patients with negative tests had endoscopies as safety‐netting, to reassure patients or if anti‐reflux surgery was being considered. Patients with an equivocal result for TFF3, atypia or p53 were recommended to be offered endoscopy.

### 2.3 Evaluation

Hospital sites uploaded data to excel databases overseen by the NHS England team. Anonymised aggregated data were provided. The pathway end was defined by latest endoscopy date or negative CS result if no endoscopy.

### 2.4 Patients Experience

Quantitative and qualitative research methodologies were used for insight on patient experience. The patient survey (Data S2) was self‐reported (*n* = 357 across 14 NHS Trusts) and adapted from the validated ENDOPREM survey instrument [17]. The sample of patients invited to interview was limited to those completing surveys, consenting for follow‐up, giving contact details and attending interviews (*n* = 28).

### 2.5 Statistics

In descriptive analyses, continuous variables were described by mean, standard deviation (SD), median, first and third quartiles (Interquartile range [IQR]: Q1, Q3). Categorical variables used number and percent. Missing data for variables were reported. Only available data were summarised; no imputation methods were used to handle missing data. Continuous outcomes were described by mean, 95% confidence intervals (CIs) (or median and interquartile range [IQR]). Binary/categorical outcomes were described by proportions and 95% CIs. Comparisons were made using the Kruskal–Wallis test. Times to event outcomes were evaluated using Kaplan–Meier (KM) methods. Median/ mean time‐to‐event were described along with 95% CIs and KM survival curves presented graphically.

## 3 Results

### 3.1 Prospective CS Cohort Analysis

**FIGURE 3:**
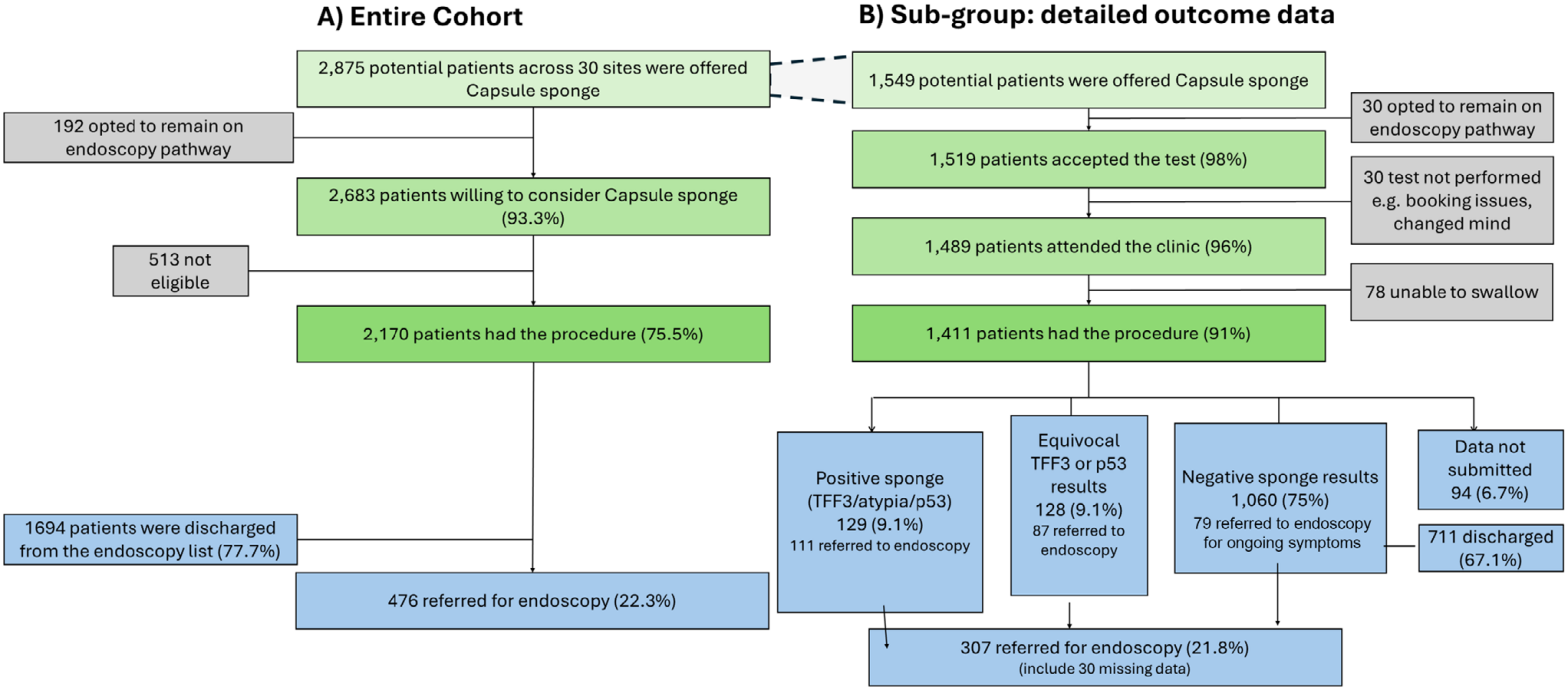
Flow diagram of the patient pathway and the overall outcomes—endoscopy or discharge for the entire cohort is shown in (A). There were a total of 2875 patients included in the entire cohort, of which 1694 were discharged and 476 had an onward endoscopy. More detailed capsule sponge and endoscopy outcomes were provided for a subgroup of patients and these outcomes are detailed in (B). A total of 1549 patients, from the 2875, had sufficient data for further investigation and detailed analysis.

In the study, 2875 patients from 23 NHS hospitals were offered CS and patient management data were returned by the trust. 2683/2875 (93.3%) agreed and 513 had exclusion criteria leaving 2170 who had a CS test. There were no serious adverse events. 476 (22%) were referred for endoscopy based on positive or insufficent CS samples and due to unresolved or new symptoms or patient anxiety. As a result 1694 (78%) were discharged from endoscopy lists (Figure 3A).

A subgroup of 1549 evaluation patients had complete outcome data submitted including in a subgroup analysis of demographic characteristics as shown (Figure 3B and Table 1). The subgroup was representative of the overall cohort, CS test was accepted in 98.1% (1519/1549), and 96.1% (1489/1549) received CS. Among patients attending, 86.9% (1294/1489) completed on first attempt and 94.8% (1411/1489) by second or third. The results’ breakdown shows 75% (1060) negative for TFF3/p53 and atypia, and 18.2% (129 + 128) had positive or equivocal results (Figure 3B).

**FIGURE 4:**
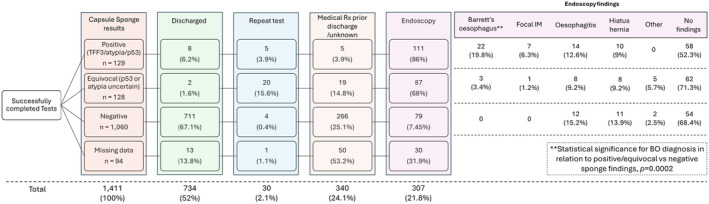
Flow diagram with detailed Capsule sponge findings and endoscopy or discharge decisions. Kruskal–Wallis test and post hoc Dunn's test, *p* = 0.0002, for Barrett's between positive and negative Capsule Sponge.

Clinical management decisions are shown in Figure 4. Of those with negative CS results, 67.1% (711/1060) were discharged. 7.5% (79/1060) were referred for endoscopy and none had BO or cancer suggesting a high specificity of a negative sponge. Discharge decisions varied considerably between sites (Table 2). More older age groups were referred for endoscopy: 36.8% (21/57) of 76+ year olds and 15.9% (80/503) 18–45‐year‐olds.

**TABLE 2:** Breakdown of number of capsule sponges completed, positive findings and onwards referral to endoscopy by hospital ‐sites commenced the evaluation project at different times.

Trust/Site	Number of Capsule sponges completed	Positive atypia or p53 requiring 2ww endoscopy	Positive TTF3 requiring endoscopy	TTF3 equivocal or N/A	Number of confirmatory endoscopies performed	Onward referral rate (%)
Barking, Havering and Redbridge (BHRUT)	57	0	2 (3.5%)	2 (3.5%)	17	29.8
Bedfordshire Hospital Trust (Bedford)	66	1	0	6 (9.1%)	37	56.1
Cambridge University Hospitals	149	1	5 (3.4%)	12 (8.1%)	42	28.2
Countess of Chester Hospital	166	2	5 (3%)	14 (8.4%)	54	32.5
Croydon Health Services NHS Trust	27	0	1 (3.7%)	7 (25.9%)	5	18.5
Cumberland Infirmary	40	0	7 (17.5%)	6 (15%)	26	65
Gloucestershire Hospital Trust	65	1	5 (7.7%)	5 (7.7%)	8	12.3
Harrogate District Trust	88	1	7 (8%)	8 (9.1%)	17	19.3
Kettering General Hospital	21	1	0	6 (28.6%)	6	28.6
Lister Hospital	319	2	30 (9.4%)	16 (5%)	71	22.3
Pennine Acute Hospitals (Royal Oldham)	16	0	1 (6.3%)	0	3	18.8
Queens Medical Centre (Nottingham)	84	0	9 (10.7%)	6 (7.1%)	15	17.9
Royal Hospital Exeter	7	0	3 (42.9%)	2 (28.6%)	1	14.3
Royal United Hospital (Bath)	65	2	0	8 (12.3%)	11	16.9
Salford Royal Hospital (Manchester)	170	3	8 (4.7%)	10 (5.9%)	11	6.5
Shrewsbury and Telford	175	3	7 (4%)	5 (2.9%)	25	14.3
St Helens Hospital	98	1	7 (7.1%)	4 (4.1%)	19	19.4
University Hospitals of Leicester	7	0	1 (14.3%)	0	0	0
University Hospitals of North Midlands (Stoke)	76	1	4 (5.3%)	2 (2.6%)	14	18.4
University Hospitals Plymouth	70	1	2 (2.9%)	5 (7.1%)	2	2.9
West Suffolk Hospital	41	0	3 (7.3%)	5 (12.2%)	14	34.1
Wigan, Wrightington and Leigh Trust	84	0	5 (6%)	4 (4.8%)	12	14.3
William Harvey Hospital (Kent)	279	1	14 (5%)	31 (11.1%)	66	23.7
Total	2170	21	149 (6.9%)	164 (7.6%)	476	22.3

Of 129 patients with positive CS, 73% had endoscopic findings of which 26% (29/111) patients had BO or IM of the proximal stomach. Barrett's was also detected following equivocal CS results (faint TFF3/p53 staining), whereas none was detected in negative CS (*p* = 0.0002) (Figure 3). Dysplasia results were not recorded but no cancers were reported. Twenty‐two patients had oesophagitis or ulceration which can lead to atypia findings on CS. Hiatus Hernia, common in reflux, was found in both groups (Figure 3). In the counterfactual group, (who were demographically similar in age, gender, ethnicity, deprivation index and referral type to the evaluation cohort‐ S3)BO rates were remarkably similar those following a positive CS (1.4%, 17/1181 counterfactual vs 1.8%, 25/1411 CS). There was therefore much higher diagnostic yield following a CS triage test since only 21.8% (307/1441) patients in the CS group required endoscopy for positive tests or symptoms, whereas on the standard pathway all patients were endoscoped. Notably most patients on routine reflux pathways had normal endoscopy (Figure 3), reinforcing the value of a triage test.

## 4 Impact on Clinical Pathway Timelines

**FIGURE 5:**
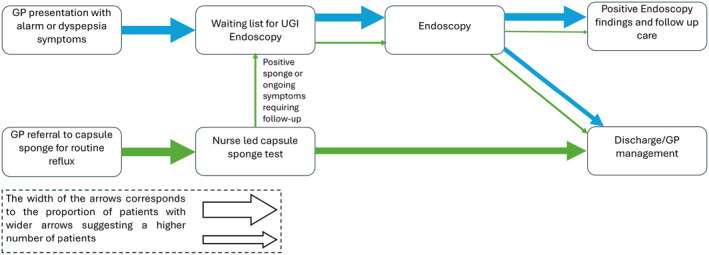
The ideal Capsule Sponge pathway, in green, and how it compares against the current clinical pathway, in blue. The width of the arrows corresponds to the proportion of patients flowing through each pathway. For example, after the nurse led capsule test, the majority of the patients are discharged or managed by their general practitioner (GP) but some are referred for an onward endoscopy.

The median (IQR) time between referral and triage date for CS was 27 (13–70) days since a nurse had to assess suitability. Among 1489 patients having CS, the median (IQR) time from triage to CS appointment was 12 (11–12) days and patients waited on average 21 days for results. Due to the two‐stage nature of the CS, the time to discharge varies depending on whether subsequent endoscopy/other tests were required. Patients with negative CS result were discharged sooner since they less frequently needed endoscopy. Urgent endoscopy after positive CS proceeded quickly (Figure 5, Table 3). The timelines for patients with negative CS results were similar to the counterfactual group, whereas a longer pathway resulted after positive CS requiring further investigation. The median time from triage to end of pathway for CS patients decreased from 55 days (95% CI: 52–67) in the early time‐period (before 31/07/2021) to 43 days (95% CI: 37–46) in later time‐periods (after 01/01/2022).

**TABLE 3:** Proportion of patients waiting to reach the end of the diagnostic pathway at different time points during the follow‐up from triage in capsule sponge and counterfactual groups.

Days	Capsule sponge patients	Capsule sponge patients (negative and then discharge)	Counterfactual patients
15	97% (96%–98%)	95% (94%–97%)	73% (67%–80%)
30	75% (73%–78%)	61% (58%–65%)	46% (39%–54%)
60	37% (35%–40%)	12% (10%–15%)	24% (18%–31%)
90	27% (25%–30%)	4% (3%–6%)	18% (13%–24%)

## 5 Patient Experience

Three hundred and fifty‐seven patients undertook patient experience surveys, and 28 were interviewed. Eighty‐two per cent (*n* = 267/357) agreed or strongly agreed that they were satisfied with their experience of CS. Patients interviewed stated that they would have the test again and recommend it to friends or family. Positive satisfaction levels were due to less invasive nature of the test, cell collection from the whole oesophagus, short appointment time and office‐based nature. Patients' reasons for not being satisfied included gagging and lack of awareness of being removed from endoscopy waiting lists.

Patient satisfaction differed by result type: higher satisfaction levels were reported more among patients having only CS (88%, 184/209) compared to patients referred to endoscopy (76%, 66/ 87). Some patients expressing preference for CS described endoscopy as painful, invasive and time‐consuming.

No serious adverse events were reported during the evaluation. Seventy‐eight per cent (*n* = 277/357) of patients reported mild or no pain. The commonest side‐effect was mild throat irritation. Those experiencing a previous endoscopy were less likely to report pain. Sixty‐two percent (*n* = 222/357) felt that their original issue was resolved “definitely” or “some extent”. Males were more likely to report that their original issue was resolved (72%) compared to females (64%).

## 6 Conclusions

The results of this large‐scale real‐world NHS evaluation show CS is feasible as a nurse‐led triage service for patients with reflux symptoms on routine/low‐risk endoscopy waiting lists. The CS pathway led to 78% discharge rate from endoscopy and had high patient acceptability, despite leading to longer clinical pathways for 22% requiring endoscopy. Endoscopic findings following a positive sponge test (TFF3/atypia/p53) were enriched for oesophagitis, BO, focal IM at the GOJ/ gastric cardia.

The offer of a CS as an alternative to endoscopy was highly accepted by patients. This was substantially higher than the BEST3 trial when 39% expressed interest in having a CS offered to individuals on reflux medication in primary care [12]. Due to variable data uploaded by sites, some bias may have been introduced. Nevertheless, the high acceptability of CS observed in the evaluation aligns with high acceptance rates observed in the NHS England Management Information (MI) data (i.e., 93%), which provided more accurate data. The invitation to have a test as part of a clinical pathway within the NHS and the impact of Covid‐19 may have influenced this higher acceptance rate.

Among patients who attended CS visits, 95% of patients had successfully completed the test, and 87% patients required only one attempt, consistent with the literature [18, 19]. Although patients were offered three attempts, it is possible that some patients made one unsuccessful swallow attempt and declined further.

The low prevalence of BO in patients on routine reflux pathways (1.8%, in the CS group and 1.4% in the counterfactual subgroups) is likely due to female predominance (58%), low age 52 (range 40–62), variation in severity in reflux history and variability in endoscopy reporting between sites. However, following a positive sponge result, this enriched substantially such that 23% (25/111) had BO diagnosed compared with no (0/79) patients with negative sponges. In comparison, in the BEST3 trial which enrolled patients > 50 years, taking acid‐suppressants for GORD for > 6 months, who had not undergone endoscopy within 5 years 8% had BO diagnosed. The referral by the GP for endoscopy of patients at very low risk for Barrett's due to their young age, especially in young women, is an interesting finding from this study, suggesting that this is an area where more referral guidance and triage testing could be particularly useful to focus resources where they are most needed in an over‐stretched service.

No cancers were identified from those having CS within the evaluation period, which underlines the low yield in routine reflux referrals and suggests that CS triage is appropriate and safe. However, there were careful safety‐netting procedures ensuring individuals with alarm symptoms or symptoms suggesting gastric pathology were not eligible for CS testing and vigilance is required in rolling out services to minimise missed cancer risks—the capsule may not pass an obstruction or cancer associated dysmotility and the stomach and duodenum cannot be assessed with CS. In addition, the CS will primarily detect Barrett's and a unique aspect to this study was the addition of p53 and atypia upfront to help detect any cancerous changes even in patients with a routine reflux referral. Furthermore, while ulcer slough or atypia of uncertain significance may indicate erosive oesophagitis this needs an endoscopy for a reliable diagnosis. This is why patients with persistent symptoms despite PPI remained on the endoscopy pathway. Additional endoscopy data and 2 year follow‐up from the largest hospital site in the evaluation have also been presented as an abstract, awaiting submission for publication and are reassuring [20].

We have shown that introduction of a CS test can be achieved efficiently in routine clinical practice with short times from test to results, and decisions to discharge or investigate. Here, all referrals were initially for endoscopy then triaged to CS and telephoned for eligibility and willingness to undergo CS. Providing CS on the GP referral forms would eliminate such delay. Among patients requiring onward endoscopy those referred for an urgent endoscopy waited 8 weeks (55 days) from their CS test, whereas those with routine referrals waited 14 weeks (100 days). Prolonged waiting times following positive tests reflect diagnostic delays but can be reduced by reducing endoscopy demand overall through triage tests such as CS and colon capsule [21, 22]. Of note, effectiveness of CS in reducing endoscopy increased over the evaluation suggesting as clinician confidence grows the impact could be substantial in terms of reducing waiting lists and furthering subsequent economic benefit.

**FIGURE 6:**
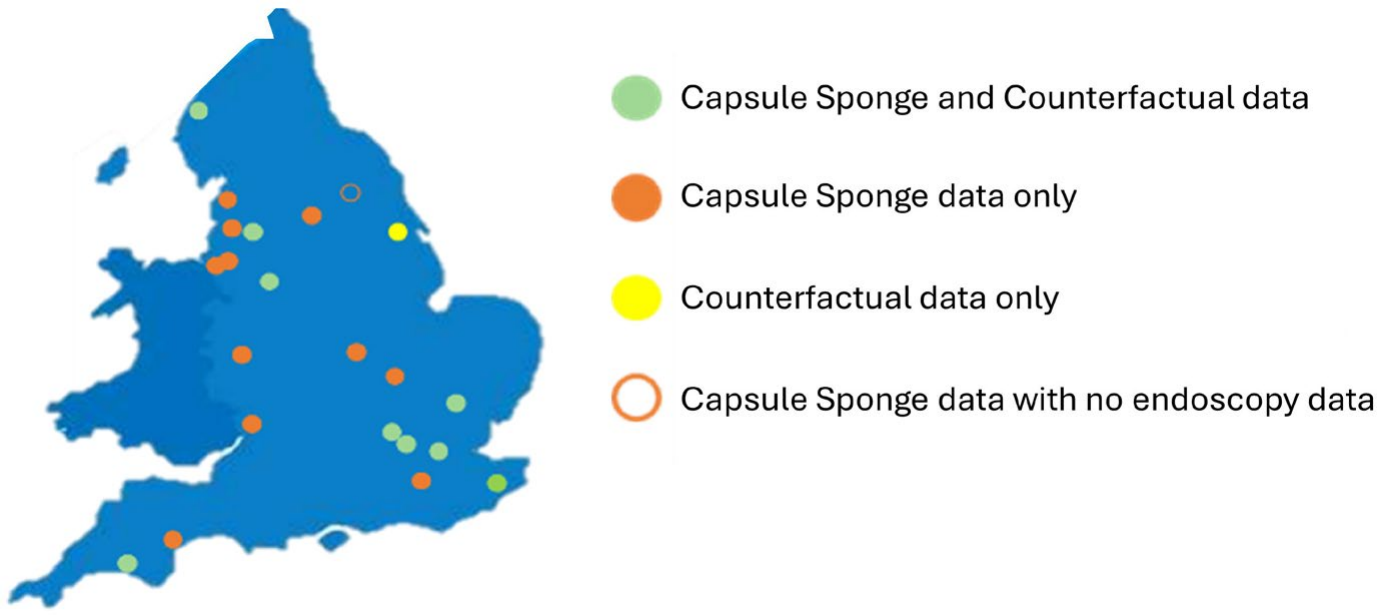
Map schematic of the areas that contributed patient data for the pilot. The green circle is for the sites that contributed both capsule sponge and counterfactual data. The orange circle demonstrates sites that contributed capsule sponge data only whereas yellow is for counterfactual data only. Finally, the orange ring shows sites that shared capsule sponge data with no endoscopy results.

This study has strengths and limitations. The strengths are the large‐scale, real‐world setting across multiple NHS sites (Figure 6). This real‐world cohort study was the next logical step following the extensive data on > 2000 patients who were enrolled in clinical trials with CS and tandem endoscopy (REF BMJ2010, PLOS MED, Lancet Oncology). The limitations are the lack of long‐term follow‐up to determine what proportion of patients discharged return into the system and any subsequent cancer diagnoses—though sites were asked to alert NHS England about any missed cancers, and none were flagged until April 2024. Continued follow‐up of this cohort will be critical to determine missed cancer rates and compare these with the counterfactual over a similar time‐period. Notably, the real‐world evaluation started during Covid‐19 when endoscopy backlogs were critical, but endoscopy and NHS pathology remain stretched. There may be considerable variation in the quality of data collected.

In conclusion, a non‐endoscopic service incorporating CS provides safe triage for patients with reflux symptoms on routine pathways, with high patient acceptability and favourable impact on reducing endoscopy waiting lists, enriching for Barrett's and expected substantial health economic benefits. Resources are required to establish services in secondary care or diagnostic hubs to build on the experience from this prospective real‐world evaluation. Longer term follow‐up is required to assess the programme impact.

## Author Contributions

**Vlasios Gourgiotis:** investigation, methodology, supervision, resources, writing – review and editing, validation, writing – original draft. **Charlotte Graham:** funding acquisition, project administration, investigation, methodology, supervision, resources, writing – review and editing. **Kristen Foerster:** methodology, investigation, funding acquisition, writing – review and editing, project administration, resources, supervision. **Rebecca C. Fitzgerald:** investigation, methodology, supervision, resources, writing – review and editing, validation, writing – original draft. **Rory Harvey:** investigation, methodology, supervision, resources, writing – review and editing. **Danielle L. Morris:** investigation, methodology, supervision, resources, validation, writing – review and editing, writing – original draft.

## Ethics Statement

All patients signed an NHS consent form and only completely anonymised and aggregated data was provided for analysis in keeping with the national Public Benefit and Privacy Panel (PBPP) approval from the Caldicott guardian.

## Conflicts of Interest

Rebecca C. Fitzgerald is named on patents for Cytosponge, which have been licensed by the Medical research Council to Covidien (now Medtronic). RCF is a co‐founder and shareholder for Cyted Ltd.

## Supporting information


**Data S1.** Clinical guidelines provided to each site by the clinical oversight committee.

**Data S2.** Cytosponge diagnostic test patient survey.

**Data S3.** Demographic data of the capsule sponge and comparable counterfactual group.

## Data Availability

The data that support the findings of this study are available from NHS England. Restrictions apply to the availability of these data, which were used under license for this study. Data are available from the author(s) with the permission of NHS England.
