# Supplementary material for: Use of a Non‐Endoscopic Capsule‐Sponge Triage Test for Reflux Symptoms: Results From the NHS England Prospective Real‐World Evaluation
Source: Aliment Pharmacol Ther. 2025 Jan 10;61(5):876–85. doi: 10.1111/apt.18472 (PMC11825927; doi:10.1111/apt.18472)
Supplement: Supplementary file 1 — Data S1. Clinical guidelines provided to each site by the clinical oversight committee. [file APT-61-876-s003.pdf]

Clinical guide for using Cytosponge in the upper GI endoscopy pathway

Scope

Cytosponge is a non-endoscopic diagnostic tool that was developed to detect Barrett's oesophagus in patients with reflux disease. It consists of a tethered sponge in a capsule that is swallowed and when brought up, it collects oesophageal cells which can be assessed for intestinal metaplasia (TFF3) and dysplasia (atypia and p53).

During the Covid-19 pandemic, endoscopy waiting lists have increased significantly in the NHS in England. Although the majority of these people will not have cancer or other serious pathology, this guidance details how Cytosponge can be used to prioritise access to upper GI endoscopy for patients referred to secondary care with reflux symptoms. Identifying those most at risk of Barrett's oesophagus and OG cancers will support services to prioritise those patients who are most at risk of serious pathology, whilst minimising the number of upper GI endoscopy procedures required.

This document provides guidance for the use of Cytosponge in the upper GI endoscopy pathway for patients referred with symptoms of gastro-oesophageal reflux. Pathological assessment of the Cytosponge samples will be carried out centrally by the company Cyted. Details of this are included in this guidance.

The NHS Cancer Programme is working with Cancer Alliances to establish Cytosponge clinics to test and develop the evidence base for this technology, in addition to supporting the restoration of endoscopy services during the Covid-19 pandemic

Clinical evidence and data on Cytosponge

- There have been three clinical trials that demonstrate the safety, acceptability and diagnostic accuracy of cytosponge to detect Barrett's Oesophagus. There are some evidence gaps for its use in secondary care, but the evidence has been deemed strong enough by the NHS Cancer Programme's Expert Advisory Group for Innovation and

the Clinical Advisory Group for a pilot roll-out. The evidence gaps will be addressed through the evaluation.

- Applicability to secondary care: the feasibility study BEST1 was conducted in 504 patients in 11 general practices [PMID: 20833740], accuracy study BEST2 performed in secondary care in >1,400 patients [PMID: 25634542] and the randomised study BEST3 involved >1,650 Cytosponges administered in 72 GP surgeries [Gastro 2019,156;S284 & PMID: 30075763]. Data from the control arm of BEST2 support the use of cytosponge in secondary care, however the data is not randomised [PMID: 25634542].

- Safety: Cytosponge administration is very safe [PMIDs: 19651633, 20833740, 25634542, 17566045, Gastro 2015, 148(4);S16]. The detachment rate is <1:5,000 with easy retrieval at endoscopy. The commonest side-effect is sore throat which resolves within a few days. It avoids the risks associated with sedation in endoscopy and many patients prefer the convenience and speed of this test compared with endoscopy. Furthermore, it can be delivered in a more Covid-secure environment

- Acceptability: The Cytosponge procedure scored a median score of 6.0 (95%CI 5.0-8.0) on a visual analogue scale from 0 (worst experience) to 10 (best experience), which is higher than endoscopy [PMIDs: 19651633, 20833740, 25634542, 17566045, Gastro 2015, 148(4);S16] and 8.6/10 in the recently completed BEST3 study [Gastro 2019,156;S284].

- Cytosponge: TFF3 test is accurate for diagnosing Barrett's oesophagus with a sensitivity of approximately 80% (intention to treat analysis) and specificity of >92%. When patients are recalled if the sample is inadequate (12-15%) the sensitivity is 94% (PMID: 25634542).

- Cytosponge can also diagnose other clinically relevant oesophageal conditions including early cancer, eosinophilic Oesophagitis (EoE), oesophageal candida, oesophagitis, and *H.pylori*, if present in the proximal stomach.

- A TFF3 positive Cytosponge indicates intestinal metaplasia (IM) - this is often from Barrett's but can also arise from IM of the gastric cardia which can indicate more extensive gastric IM, a premalignant condition. In the BEST3 study this has led to an early diagnosis of gastric cancer in one case.

- Cost-effectiveness: A full health economic analysis is underway for BEST3 and cost effectiveness and value for money will be looked at as part of the evaluation of this pilot.

Target population and exclusions

This guidance is specific for the use of Cytosponge in patients referred with reflux symptoms with no alarm symptoms by general practitioners to secondary care on a diagnostic upper GI endoscopy pathway.

Inclusion criteria for Cytosponge:

- Patients with symptoms of reflux including:
  - Heartburn (burning sensation on chest usually after eating),
  - Regurgitation (an unpleasant sour taste in mouth caused by stomach acid)
  - Waterbrash (excessive salivation)

Exclusion criteria for Cytosponge (absolute contraindications):

- **Alarm symptoms:**
  - **dysphagia**
  - **dyspepsia and weight loss**
  - **dyspepsia and anaemia**
- Previous cancer of the oesophagus
- Patient with a diagnosis of an oropharyngeal, oesophageal or gastro-oesophageal tumour
- Patient who has had treatment to the oesophagus e.g. photo dynamic therapy, endoscopic mucosal resection, radio frequency ablation, surgery
- Patient known to have oesophageal varices or cirrhosis of the liver
- Patient with a known anomaly of the oesophagus e.g. webbing, pouch, stricture etc.
- Patients who are pregnant (relative contraindication, Cytosponge not harmful but may not be appropriate)
- Patients unable to give consent
- Patients who have had a stroke or any other neurological disorder where their swallowing has been affected
- Patients who have had a myocardial infarction in the last 3 months.

Patients to consider as having relative contra-indications to Cytosponge use:

- Patients who have had fundoplication may be candidates for Cytosponge but may have reflux symptoms post procedure.

Triaging process

A Dyspepsia Nurse or Gastroenterologist will identify appropriate patients referred to secondary care for endoscopy that meet the criteria for the Cytosponge test. A triaging appointment will be carried out by the nurse over the phone to check their eligibility. A specified triage assessment form (provided nationally) should be completed.

Suitable patients should be invited for a Cytosponge test and provided with a patient leaflet (paper or online).

Preprocedural preparation

Patients may require a Covid-19 swab prior to the Cytosponge test being performed, please follow your Trust's local endoscopy pathway guidance

Patients should be advised to be nil by mouth 4 hours prior to the appointment for the Cytosponge test. For patients on anticoagulant medication, specific instructions should be provided.

Anticoagulation

For patients on anticoagulation therapy, please follow the appropriate guidance below, which aligns to the BSG guidance on low risk endoscopy procedures. Please note, this guidance is different to the practice carried out in the BEST trials.

<u>P2Y12 Receptor Antagonist Antiplatelet Agents e.g. Clopidogrel, Prasugrel, Ticagrelor</u>

- Continue therapy

<u>Direct Oral Anticoagulants e.g. Dabigatran, Rivaroxaban, Apixaban, Edoxaban</u>

- Omit DOAC on morning of procedure

<u>Warfarin</u>

- Continue warfarin

- Check INR during the week before endoscopy
    - If INR within therapeutic range, continue daily dose
    - If INR above therapeutic range (above 3.5), escalate to their responsible physician and reduce daily dose until INR returns to therapeutic range

Consent

This should be undertaken in the same way as consent is obtained for upper GI endoscopy i.e. the completion of an NHS Consent Form 1 and retained in the patient notes. If the patient is willing for their anonymised sample and data to be used for research, then the research box should be ticked.

Procedure

The Cytosponge may be performed by a trained nurse or healthcare professional that has been assessed and deemed competent to carry out the procedure independently (see below for training requirements).

Before the procedure, please check whether the patient has any swallowing difficulties. If the patient has any dysphagia, they are not eligible for Cytosponge and an endoscopy should be carried out.

Before using a Cytosponge, the device should be inspected, and the expiration date should be checked. Do not use the device if it is damaged or it has expired. If a device looks damaged, such as visible cracks in the capsule or protrusion of the sponge through the capsule, it should be reported to Medtronic by contacting Sarah.Butler@Medtronic.com.

The patient is asked to sit in a chair and swallow the Cytosponge capsule and string together with some water. The end of the string is attached to a piece of card which the nurse will hold. The capsule will be in the patient’s stomach for around 7½ minutes until it dissolves completely, releasing the sponge inside the stomach. The nurse will then remove the sponge from the patient’s stomach and up through the oesophagus by pulling quickly and gently on the string taking about 1-2 seconds. The patient has the option to have a local anaesthetic spray into their throat before the sponge is removed (figure 1).

The sponge is then placed in a sample collection kit provided by Cyted and secured with a patient identification label which includes the hospital number, year of birth and sex of the

patient. The sample collection kit is accompanied by a patient requisition/request form which should be completed during the clinic. Patient specimens and requisition forms are shipped using a secure courier network to Cyted who will carry out the pathological assessment. Diagnostic results are shared with the requesting clinician using NHS.net emails or an electronic reporting connector within 14 working days from the day of sample receipt in the lab.

In the rare event (less than 1/5000), that the Cytosponge detaches and is retained in the stomach, urgent upper GI endoscopy and removal of Cytosponge must be performed. Please follow local guidance and pathways for removal of foreign body.

In the extremely unlikely event that there is inhalation of the Cytosponge (never happened to date), urgent consultation with interventional respiratory or cardiothoracic teams is required and urgent bronchoscopy will be required.

Please note the following factors when you withdraw the sponge:

- **Blood**. If there is any blood on the sponge this could indicate severe inflammation or cancer. The patient should be referred for endoscopy as urgent two-week wait.
- **Lax string**. You should feel some tension when you withdraw the sponge even if there is a hiatus hernia. A lax string on withdrawal is usually from a poor swallow and therefore may mean the result is inadequate. Please note this on the Cyted request form.

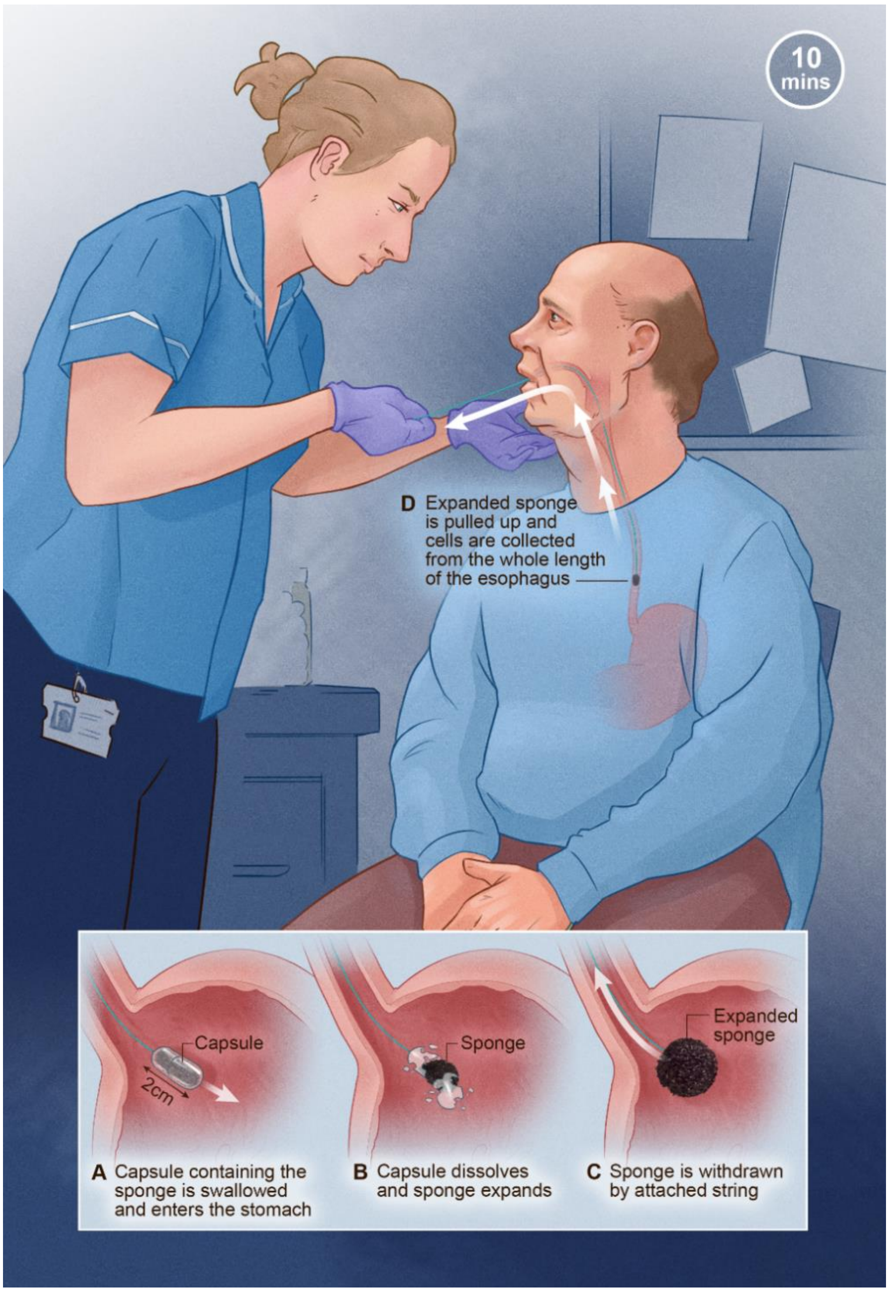

Figure 1: Administration and passage of the Cytosponge device to obtain a sample of oesophageal epithelial cells. Drawn by Campbell Medical Illustration (Glasgow, Scotland)

Discharge and safety netting

Following the procedure, the patient will be given instructions regarding follow up arrangements and who to contact if there is a query or problem post procedure. The patient will be advised that the results will be available in approximately 2-3 weeks' time, and this will be communicated by letter and/or telephone by the hospital trust. The report received from Cyted must be formally uploaded onto the patient records. The hospital trust is also

responsible for informing the GP or referring clinician via letter of the patient outcome and what follow-up plan has been arranged.

Common symptoms post procedure

The most common side effect of the procedure is a sore throat, and this can be treated with appropriate conservative measures or simple analgesia. Mild abdominal pain and nausea may also be experienced.

Symptoms post procedure that require urgent clinical review

Symptoms such as black stool, significant chest pain, severe throat pain, persistent difficulty swallowing, abdominal pain and difficulty breathing require urgent assessment. If a patient develops these symptoms immediately following the procedure, an assessment should be made urgently by the most senior physician. If a patient develops these symptoms following discharge at home, they should be advised to seek urgent medical attention in their local Accident and Emergency department.

Further follow up will be organised for the patient according to the results (see figure 2). Please correlate results with clinical symptoms and manage accordingly.

Cytopathology results

- Atypia (definite or of uncertain significance) and/or abnormal or equivocal p53. Patient requires endoscopy as urgent two-week wait.

TFF3 results

- TFF3 negative + normal P53, no atypia. Patient managed for dyspepsia according to local trust guidance with follow-up arranged by telephone with safety netting. Current symptomatic management may be continued.
- TFF3 positive **only** (+normal P53, no atypia). Patient requires routine endoscopic assessment.
- TFF3 Equivocal for Intestinal Metaplasia, Normal P53, no atypia. Patient requires repeat Cytosponge testing in 6-12 months.

Inadequate samples

- Insufficient sample: Patient requires repeat Cytosponge testing or an endoscopy as soon as feasible (within 3 months).
- Squamous cells only, no atypia: This suggests that the device did not reach the stomach. Patient probably requires repeat Cytosponge testing or an endoscopy if Barrett's or gastro-oesophageal junction pathology needs to be excluded.

Other findings

- Other benign diagnoses/findings e.g. inflammation, candida etc. Patient treated according to clinical judgement

Patients with a negative test result but with ongoing symptoms may be followed-up with an appointment in a dyspepsia clinic where this is locally available or may need to be re-referred for further investigation. This appointment should discuss the management of ongoing symptoms as part of safety netting.

Figure 2: Follow up required for Cytosponge sample results including endoscopy guidance where required

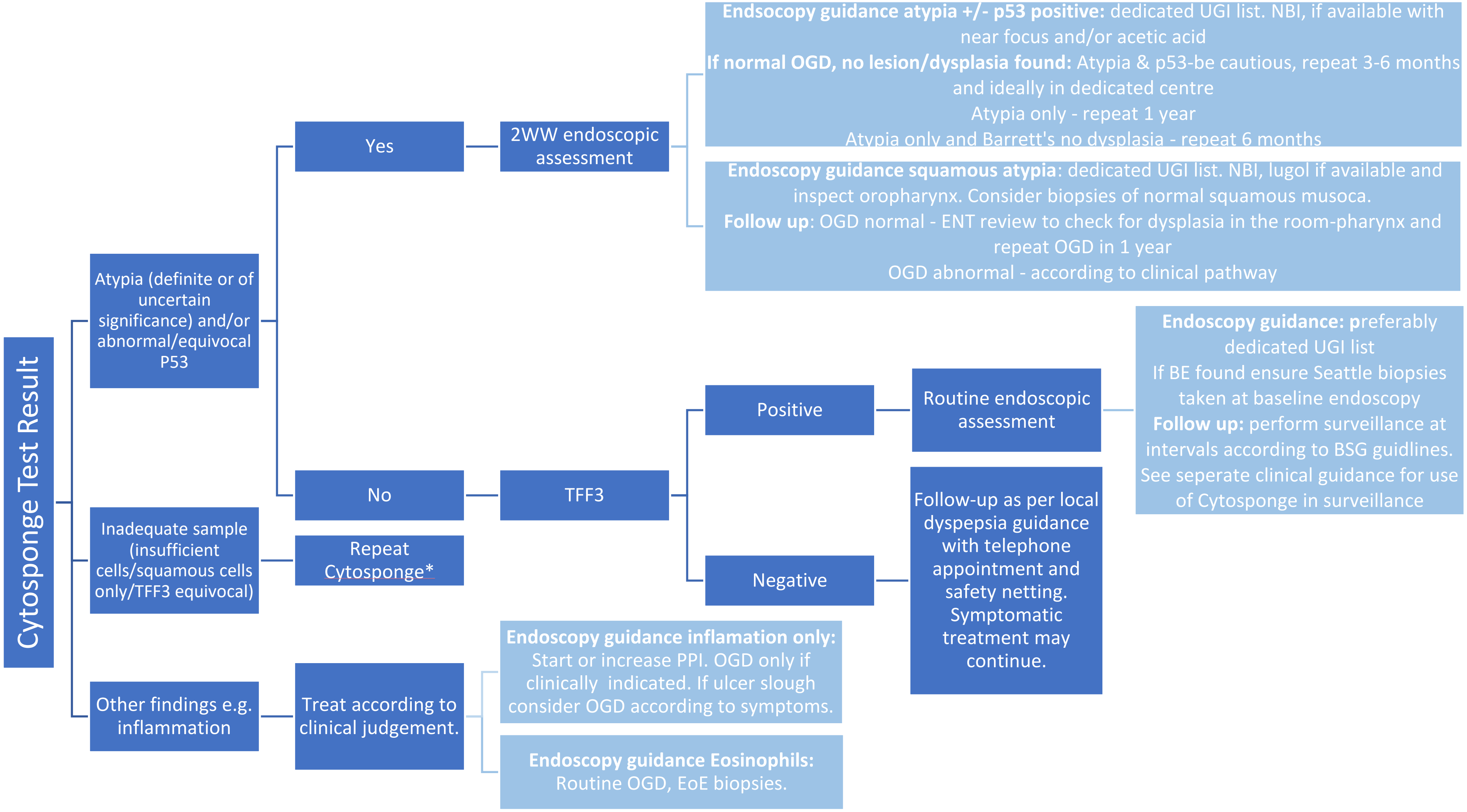

Oesophagus-Gastro-Duodenoscopy (OGD) protocol for TFF3+ Cytosponge

- TFF3 is an intestinal metaplasia biomarker not just a Barrett's marker
- Use narrow-band imaging endoscopy where possible.
- Pay special attention to the distal oesophagus and gastro-oesophageal junction (GOJ) for visible Barrett's, an irregular z-line which may have more focal intestinal metaplasia.
- Number of gland groups positive for TFF3 are indicative: ≥ 3 expect Barrett's, < 3 expect irregular z-line/ gastric intestinal metaplasia.

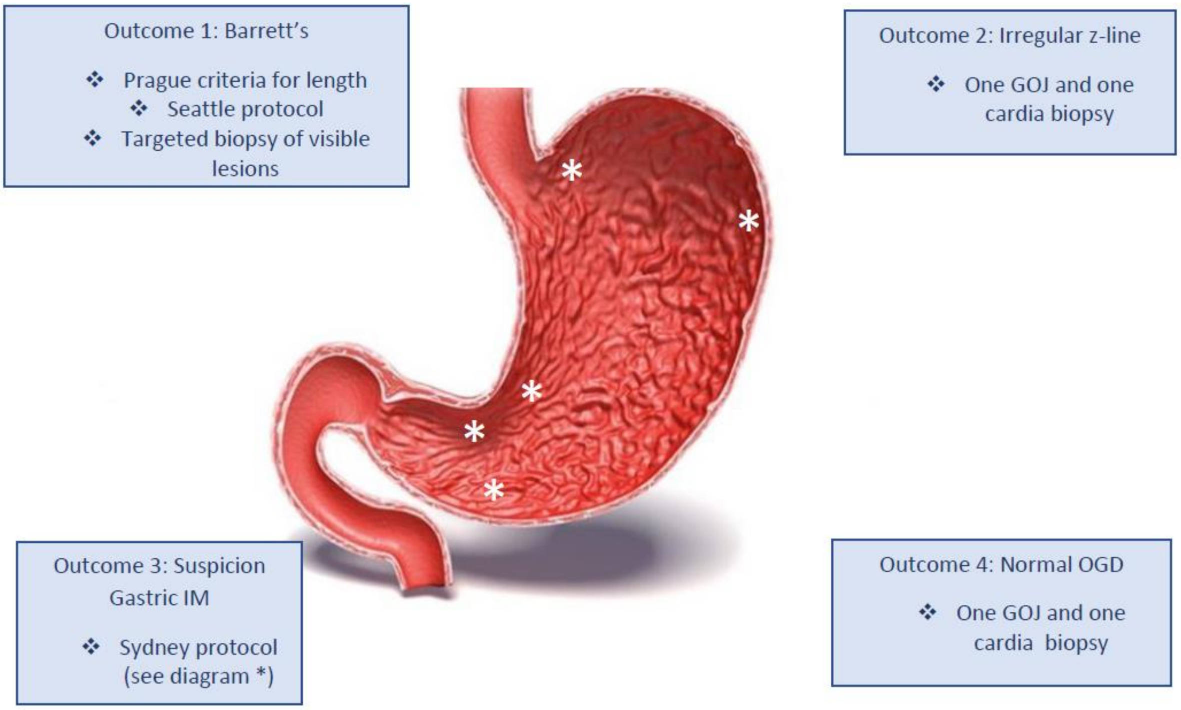

Seattle protocol: 4-quadrant biopsies of every 2cm of Barrett's

Sydney protocol: 2 biopsies of the antrum of the lesser and greater curvature (in 1 cassette), 2 biopsies of the body of the lesser and greater curvature (in 1 cassette), 1 biopsy of the incisura

Training

Training will be organised nationally and delivered locally on a one-to-one basis in individual hospital trusts. Clinical leads in each pilot clinic are responsible for ensuring nurses (band 7+ or band 6 if supported by band 7+) with sufficient experience are nominated to undertake training. Training may be completed within one clinic session.

All staff delivering Cytosponge procedures must have completed and passed the training and competency assessment.

Evaluation

Hospitals participating in this pilot will be expected to collect a minimum dataset for each procedure including patient outcomes, which will feed into a national service evaluation delivered by an external evaluator and managed by the NHS Cancer Programme. All Trusts must nominate a Data Collection Lead for the Cytosponge pilot.

The aim of the evaluation is to understand the impact of Cytosponge on (1) endoscopy demand, (2) patient outcomes, (3) diagnostic experience and (4) patient inequalities. There will also be a process evaluation and economic evaluation.
