# Supplementary material for: Use of a Non‐Endoscopic Capsule‐Sponge Triage Test for Reflux Symptoms: Results From the NHS England Prospective Real‐World Evaluation
Source: Aliment Pharmacol Ther. 2025 Jan 10;61(5):876–85. doi: 10.1111/apt.18472 (PMC11825927; doi:10.1111/apt.18472)
Supplement: Supplementary file 2 — Data S2. Cytosponge diagnostic test patient survey. [file APT-61-876-s002.pdf]

CYTOSPONGE DIAGNOSTIC TEST – PATIENT SURVEY

This survey is about your experiences of the Cytosponge (sponge on a string) diagnostic test. This is an opportunity to feedback on your experience whether you chose to attend your appointment or not. We want to know more about your experiences of having the test, or your reasons for deciding not to have the test. Your views about the Cytosponge diagnostic test will help improve the quality of this service in the future. This will include both services provided by the hospital which invited you for a Cytosponge test and services provided by others.

What to do
For each question, please click inside the box that is closest to your views. If you make a mistake, simply click the correct box and the system will update automatically. If you cannot or do not want to answer a question, leave it blank and continue.

Completing the survey should take around 15-20 minutes.

Important information
Under data protection legislation, NHS England (NHSE) is the controller responsible for your personal data used to conduct the survey. IQVIA is the Data Processor and has been appointed by NHSE to securely administer this survey.

By completing this survey, you are giving consent that the information you provide can be used by IQVIA to evaluate the patient experience of the Cytosponge test. Any research teams analysing the data will be subject to strict rules for the security and confidentiality of your information. Your responses will not be shared with others unless required by law or where there is a clear overriding public interest. Your responses will be destroyed 3 months after the final report has been provided to NHSE.

Your responses to this survey will be handled securely and confidentially, and the results published will not identify you. IQVIA will combine your responses with the other responses to summarise the data and share a report with NHSE. Where any comments are made, these will be looked at in full by researchers analysing the data but IQVIA will remove any information that could identify yourself or others before passing them on to NHSE, unless there is a need to escalate an issue of concern.

At the end of the questionnaire, you will be asked if you would like to participate in a follow up interview. If you provide your name and address here, these details will only be used for the purpose of inviting you to an interview. Researchers may refer back to responses you have given in this questionnaire.

You can withdraw the information you give in the survey upon request, up to the point at which the data is analysed and aggregated. If you would like to discuss deletion of your data, you can contact the survey helpline at IQVIA.

If you have any queries about the survey, how your information may be used or if you wish to opt out, please call the FREEPHONE helpline number on 0800 783 1775 or email: helpline-QH@iqvia.com

This questionnaire was developed with support from The Newcastle ENDOPREM™: a validated patient reported experience measure for gastrointestinal endoscopy. Please see https://pubmed.ncbi.nlm.nih.gov/34697041/ for more.

About you

1. What year were you born?

(Please write in) e.g. | 1 | 9 | 4 | 4 |

Y	Y	Y	Y

2. At birth, were you registered as	
Male	☐
Female	☐
Intersex	☐
I would prefer not to say	☐

3. Is your gender identity the same as the sex you were registered at birth?	
Yes	☐
No	☐
I would prefer not to say	☐

4. To which of these ethnic groups do you belong? (Tick ONE only)					
WHITE					
1 ☐	English / Welsh / Scottish / Northern Irish / British	2 ☐	Irish	3 ☐	Gypsy or Irish traveller
4 ☐	Any other white background (Please write in box)				
MIXED					
5 ☐	White and black Caribbean	6 ☐	White and black African	7 ☐	White and Asian
8 ☐	Any other mixed background (Please write in box)				
ASIAN OR ASIAN BRITISH					
9 ☐	Indian	10 ☐	Pakistani	11 ☐	Bangladeshi
12 ☐	Chinese	13 ☐	Any other Asian background (Please write in box)		
BLACK OR BLACK BRITISH					
14 ☐	African	15 ☐	Caribbean		
16 ☐	Any other Black background (Please write in box)				
OTHER ETHNIC GROUP					
17 ☐	Arab	18 ☐	Any other ethnic group (Please write in box)		

Introduction

5. After being offered a Cytosponge test, did you take up the offer and arrange an appointment to receive the test?	
1☐ Yes ➔ Go to question 7	2☐ No ➔ Go to question 6

[If no response, exit questionnaire]

6. Why did you **not** take up the offer to have the Cytosponge test? Please tick all that apply	
I did not like the sound of how the Cytosponge test is carried out ➔ Go to question 11	☐
I preferred to have an endoscopy ➔ Go to question 11	☐
I thought I was invited by mistake ➔ Go to question 11	☐
My symptoms resolved on their own ➔ Go to question 11	☐
I was not given enough information to make an informed decision ➔ Go to question 11	☐
Other (Please tell us more in the box below) ➔ Go to question 11	☐

[If no response, route to Q11]

7. Did you attend your Cytosponge test?	
1☐ Yes ➔ Go to question 9	2☐ No ➔ Go to question 8

8. Why did you **not** attend your Cytosponge test appointment? Please tick all that apply	
Due to COVID-19 e.g. isolating, shielding, concerned about travelling to hospital, worried about the virus, waiting to be vaccinated	☐
I did not like the sound of how the Cytosponge test is carried out ➔ Go to question 11	☐
I preferred to have an endoscopy ➔ Go to question 11	☐
I thought I was invited by mistake ➔ Go to question 11	☐
My symptoms resolved on their own ➔ Go to thank you message and save	☐
Unable to attend the appointment e.g. due to travel, illness, or other reasons ➔ Go to question 11	☐
I did not understand the invitation materials ➔ Go to question 11	☐
Other (Please tell us more in the box below) ➔ Go to question 11	☐

[If no response, route to Q11]

9. When did you have the Cytosponge test?	
Less than 3 months ago	☐
4-6 months ago	☐
7-9 months ago	☐
More than 9 months ago	☐
Don't know / can't remember	☐

10. How long did you wait from when you were referred by your GP for further tests to investigate your reflux symptoms and your Cytosponge appointment?	
Less than one month	☐
Between 1-3 months	☐
Between 3-6 months	☐
Between 6-12 months	☐
Over 12 months	☐

Upper endoscopy / gastroscopy history - ***(to be asked to all respondents)***

11. Prior to being offered a Cytosponge test, had you ever had an upper endoscopy or gastroscopy test (Camera or tube inserted through the mouth or nose into the stomach)?	
Yes, one	☐
Yes, two	☐
Yes, three	☐
Yes, more than three	☐
No ➔ Go to question 13	☐
Don't know / can't remember ➔ Go to question 13	☐

[Respondents answering No at Q5 and No or Don't know/can't remember at Q11 should exit here]

12. How long ago was your last upper endoscopy or gastroscopy (not including an upper endoscopy or gastroscopy you had following a Cytosponge test)?	
Less than a year	☐
1-2 years	☐
3-5 years	☐
Over 5 years	☐
Don’t know / can’t remember	☐

If respondents answered No to Q5, at this point they should be sent to the thank you message and save.

<u>Before coming for your test -</u> ***(only to be asked to those who said Yes to Q5)***

In this section, we want to find out about the time leading up to your test/s, before you came to hospital.

13. To help you make your decision, were you told that if you chose a Cytosponge test, that you would be taken off the waiting list for an upper endoscopy/ gastroscopy?		
1☐ Yes	2☐ No	3☐ Don’t know / can’t remember

14. Before you made your decision, did you understand the explanation of what the Cytosponge test was and what would happen during the test?	
Yes, definitely	☐
Yes, to some extent	☐
No	☐
Don’t know / can’t remember	☐

15. Before you made your decision, was it explained what would happen to you after you got the results of the Cytosponge test?	
Yes, definitely	☐
Yes, to some extent	☐
No	☐
Don’t know / can’t remember	☐

16. Please tell us how much you agree or disagree with the following statement:
I understood why I was offered a Cytosponge test.

Strongly agree ➔ Go to question 18	☐
Agree ➔ Go to question 18	☐
Neither agree nor disagree ➔ Go to question 17	☐
Disagree ➔ Go to question 17	☐
Strongly disagree ➔ Go to question 17	☐

[If no response, go to question 18]

17. Please can you tell us a bit more about what you did not understand?

Please note that the comments you provide will be looked at in full by NHS England and researchers analysing the data. Any information you give that could identify anyone will only be used if there are areas of concern. We will remove any information that could identify you before publishing any of your feedback.

18. Please tell us how much you agree or disagree with the following statement:
I had enough time to discuss the Cytosponge test with the person who invited me.

Strongly agree	☐
Agree	☐
Neither agree nor disagree	☐
Disagree	☐
Strongly disagree	☐

19. Were you given the Cytosponge patient information leaflet?

Yes, before my appointment for the test	☐
Yes, at my appointment for the test	☐
No ➔ Go to question 21	☐
Don’t know / can’t remember ➔ Go to question 21	☐

20. Thinking about the Cytosponge patient information leaflet, how much do you agree or disagree with the following statements?							
**Please tick one box on each line**.	Strongly agree	Agree	Neither agree nor disagree	Disagree	Strongly disagree	Don't know/ can't remember	Not applicable
I found the Cytosponge leaflet useful to read.	☐	☐	☐	☐	☐	☐	☐
After reading the Cytosponge leaflet, I did not have any questions about the test.	☐	☐	☐	☐	☐	☐	☐
I was able to get the information about the Cytosponge test in another language or another format e.g. large print if I needed it?	☐	☐	☐	☐	☐	☐	☐

21. Please tell us how much you agree or disagree with the following statement: **I was given enough information in order to make an informed decision about taking up the offer of a Cytosponge test?**	
Strongly agree	☐
Agree	☐
Neither agree nor disagree	☐
Disagree	☐
Strongly disagree	☐

[If respondents answered No to 7, at this point they should be sent to the thank you message and save.]

<u>During the Cytosponge test -</u> ***(only to be asked to those who said they had the test in Q7)***

In this section we would like to know about your experience of the test, from arriving in the test room until it was time to leave.

22. Please tell us how much do you agree or disagree with the following statements?

**Please tick one box on each line**.	Strongly agree	Agree	Neither agree nor disagree	Disagree	Strongly disagree	Don't know/ can't remember
I was satisfied with the explanation given to me about the test by the person carrying out the test.	☐	☐	☐	☐	☐	☐
I felt confident that the person carrying out the test knew what they were doing.	☐	☐	☐	☐	☐	☐
The person doing the test addressed any concerns I had.	☐	☐	☐	☐	☐	☐

23. Did you experience any of the following reactions during or immediately after the test?

**Please tick one box on each line**.	Not at all	Mild	Severe	Very severe	Don't know/ can't remember
Pain	☐	☐	☐	☐	☐
Discomfort	☐	☐	☐	☐	☐
Bleeding	☐	☐	☐	☐	☐
Throat irritation	☐	☐	☐	☐	☐
Other please specify	☐	☐	☐	☐	☐

24. Did the person carrying out the Cytosponge test explain how you might feel in the following days after the test?

Yes, definitely	☐
Yes, to some extent	☐
No	☐

Don’t know / can’t remember ☐

25. Did the person carrying out the Cytosponge test tell you who to contact if you were worried about **discomfort** following the test?	
Yes	☐
No	☐
Don’t know / can’t remember	☐

26. Please tell us how much you agree or disagree with the following statement: **During the test, my dignity was maintained at all times**.	
Strongly agree	☐
Agree	☐
Neither agree nor disagree	☐
Disagree	☐
Strongly disagree	☐
Don’t know / can’t remember	

27. Please tell us how much you agree or disagree with the following statement: **When I left my appointment, I was clear about what the next steps would be**.	
Strongly agree	☐
Agree	☐
Neither agree nor disagree	☐
Disagree	☐
Strongly disagree	☐
Don’t know / can’t remember	

After the Cytosponge test - ***(only to be asked to those who said they had the test in Q7)***

In this section we would like to know about your experience after the Cytosponge test including the results, if you've had them.

28. In the days following your Cytosponge test did you experience any discomfort?					
1☐	Yes – Please specify what discomfort you experienced below	2☐	No ➔ Go to question 30	3☐	Don’t know / can’t remember ➔ Go to question 30

29. Did you report the discomfort you experienced?					
1☐	Yes – Please specify who you reported it to below	2☐	No	3☐	Don't know / can't remember

30. Have you received the results of your Cytosponge test?					
1☐	Yes	2☐	No<br>➔ Go to question 36	3☐	Don't know / can't remember<br>➔ Go to question 36

31. How were you told your results?	
Letter (post)	☐
Email	☐
Phone	☐
In person	
Other - Please specify	☐

32. How long after your test did you receive your results?	
Less than 2 weeks	☐
Between 2 and 3 weeks	☐
Between 3 and 4 weeks	☐
Between 4 and 5 weeks	☐
More than 5 weeks	☐

33. Please tell us how much you agree or disagree with the following statements?

**Please tick one box on each line**.	Strongly agree	Agree	Neither agree nor disagree	Disagree	Strongly disagree	Don't know / can't remember
I understood the results of the Cytosponge test when I received them.	☐	☐	☐	☐	☐	☐
After receiving my results, I was clear about what the next steps would be.	☐	☐	☐	☐	☐	☐

34. Following your Cytosponge test, were you referred for a subsequent camera diagnostic test, like an upper endoscopy or a gastroscopy?

1☐ Yes 2☐ No 3☐ Don't know / can't remember

35. Please tell us how much you agree or disagree with the following statement:
Following my Cytosponge test and receiving my results, I was able to access advice and support from health professionals if I needed it.

Strongly agree	☐
Agree	☐
Neither agree nor disagree	☐
Disagree	☐
Strongly disagree	☐
Don't know / can't remember	☐
Not required / not applicable	☐

Overall experience - ***(only to be asked to those who said they had the test in Q7)***

36. Do you feel that the original issue (for which you were referred to hospital) has been resolved as far as possible?

Yes, definitely	☐
Yes, to some extent	☐
No	☐
Don't know / can't remember	☐

37. Please tell us how much you agree or disagree with the following statements.

**Please tick one box on each line**.	Strongly agree	Agree	Neither agree nor disagree	Disagree	Strongly disagree	Don’t know / can’t remember
I was happy to be offered/receive a Cytosponge test instead of waiting for an upper endoscopy/gastroscopy.	☐	☐	☐	☐	☐	☐
Overall, I was satisfied with my experience of the Cytosponge test.	☐	☐	☐	☐	☐	☐

38. Please can you tell us why you gave this answer

Please note that the comments you provide will be looked at in full by NHS England and researchers analysing the data. Any information you give that could identify anyone will only be used if there are areas of concern. We will remove any information that could identify you before publishing any of your feedback.

39. If there is something else you would like to tell us about your Cytosponge test, please use the space below.

Please note that the comments you provide will be looked at in full by NHS England and researchers analysing the data. Any information you give that could identify anyone will only be used if there are areas of concern. We will remove any information that could identify you before publishing any of your feedback.

Consent to interview
(only to be asked to those who said they had the test in Q7)

40. Your responses to this survey provide valuable feedback which will contribute to how the NHS improves and plans future services for patients. To better understand the patient experience, IQVIA may carry out follow-up interviews with patients to explore experiences of receiving a Cytosponge test. If you are interested in participating in a follow-up interview, please complete the following questions. IQVIA will process your responses in confidence. Your details will only be used to invite you to take part in a follow-up interview. Your details will not be shared with anyone else for any other purpose.For more information on IQVIA stores and processes your information please visit: https://www.iqvia.com/about-us/privacy. Your details will only be held for 3 months after the end of this evaluation, after which it will be securely deleted.
1 ☐ **Yes, I consent** to be contacted for a follow-up interview ➔ Go to question 41   2 ☐ **No, I do not consent** to be contacted for a follow-up interview ➔ Thank you message and save

41. Please provide your name and select if you are happy for us to contact you in the following ways	
Name:	
By email	☐
Please enter your email address:	
By post	☐
Please enter your address:	
By telephone:	☐
Please enter your telephone number:	

Thank you for taking part in the Cytosponge patient experience survey. Your input is hugely appreciated.
