# Supplementary material for: Use of a Non‐Endoscopic Capsule‐Sponge Triage Test for Reflux Symptoms: Results From the NHS England Prospective Real‐World Evaluation
Source: Aliment Pharmacol Ther. 2025 Jan 10;61(5):876–85. doi: 10.1111/apt.18472 (PMC11825927; doi:10.1111/apt.18472)
Supplement: Supplementary file 3 — Data S3. Demographic data of the capsule sponge and comparable counterfactual group. [file APT-61-876-s001.pdf]

Supplement 3 - Demographic data of the Capsule Sponge and comparable Counterfactual group

Variable	Capsule Sponge group (N=1338)*	Counterfactual group (N=289)**
**Age at referral (Median, IQR)**	53.00 (40.00-63.00)	56.00 (42.00-67.00)
**Female (n, %)**	773 (57.8%)	175 (60.6%)
**Ethnicity (n, %)**		
White	1229 (91.9%)	276 (95.5%)
Black, Black British, Caribbean or African	17 (1.3%)	1 (0.4%)
Asian or Asian British	53 (4.0%)	2 (0.7%)
Mixed or Multiple Ethnic	18 (1.4%)	1 (0.4%)
Other	21 (1.6%)	9 (3.1%)
**IMD quintile**		
1 – Most deprived	264 (19.7%)	70 (24.2%)
2	248 (18.5%)	68 (23.5%)
3	259 (19.4%)	61 (21.1%)
4	268 (20.0%)	53 (18.3%)
5 – Least deprived	299 (22.4%)	37 (12.8%)
**Type of referral**		
Direct access	227 (17.0%)	61 (21.1%)
Routine	1111 (83.0%)	228 (78.9%)
**Calendar time of referral (quarter)**		
Q2 2020	3 (0.2%)	1 (0.4%)
Q3 2020	6 (0.5%)	7 (2.4%)
Q4 2020	61 (4.6%)	6 (2.1%)
Q1 2021	149 (11.1%)	75 (26.0%)
Q2 2021	230 (17.2%)	120 (41.5%)
Q3 2021	321 (24.0%)	41 (14.3%)
Q4 2021	345 (25.8%)	22 (7.6%)
Q1 2022	223 (16.7%)	17 (5.9%)
